# A Directory for the Dissection of the Human Body

**Published:** 1877-07

**Authors:** 


*A Directory for the Dissections of the Human Body.* By JOHN CLELAND, M. D., F. R. S., Professor of Anatomy and Physiology in Queen's College, Galway. Philadelphia: Henry C. Lea. 1877. Buffalo, T. H. Butler.

For practical use in the dissecting room, this small and unpretending work will be found to be of vastly more importance than many of the works which under the name of dissectors aspire to the dignity of text books on anatomy, if one were to judge from their contents.

The author in his preface tells us that the object of this work is to guide the student in his dissections; first, that he may display the anatomy to the best advantages; second, that he may recognize the structures by their anatomical names; and all who may have occasion to refer to it, must acknowledge that these objects are fully attained.

We cannot too highly recommend this to the junior dissector. In it he will find assistance not to be found in the various works on anatomy, which are commonly used for reference in dissecting. C.

